# A scoping review evaluating physical and cognitive functional outcomes in cancer survivors treated with chemotherapy: charting progress since the 2018 NCI think tank on cancer and aging phenotypes

**DOI:** 10.1007/s11764-024-01589-0

**Published:** 2024-05-14

**Authors:** Mostafa Mohamed, Mustafa Ahmed, AnnaLynn M. Williams, Nikesha Gilmore, Po-Ju Lin, Sule Yilmaz, Marielle Jensen-Battaglia, Karen Mustian, Michelle Janelsins, Supriya Mohile

**Affiliations:** 1 Department of Medicine, University of Rochester, Rochester, NY USA; 2 Department of Surgery, University of Rochester, Rochester, NY USA; 3 Department of Public Health, University of Rochester, Rochester, NY USA; 4 Wilmot Cancer Institute, 601 Elmwood Avenue, Box 702, Rochester, NY 14642 USA

**Keywords:** Cancer survivors, Aging phenotypes, Accelerated aging, Physical function, Cognitive function, Chemotherapy

## Abstract

**Purpose:**

The primary goal of this scoping review was to summarize the literature published after the 2018 National Cancer Institute think tank, “Measuring Aging and Identifying Aging Phenotypes in Cancer Survivors,” on physical and cognitive functional outcomes among cancer survivors treated with chemotherapy. We focused on the influence of chemotherapy on aging-related outcomes (i.e., physical functional outcomes, cognitive functional outcomes, and frailty), given the known associations between chemotherapy and biologic mechanisms that affect aging-related physiologic processes.

**Methods:**

A search was conducted across electronic databases, including PubMed, Scopus, and Web of Science, for manuscripts published between August 2018 and July 2023. Eligible studies: 1) included physical function, cognitive function, and/or frailty as outcomes; 2) included cancer survivors (as either the whole sample or a subgroup); 3) reported on physical or cognitive functional outcomes and/or frailty related to chemotherapy treatment (as either the whole sample or a subgroup); and 4) were observational in study design.

**Results:**

The search yielded 989 potentially relevant articles, of which 65 met the eligibility criteria. Of the 65 studies, 49 were longitudinal, and 16 were cross-sectional; 30 studies (46%) focused on breast cancer, 20 studies (31%) focused on the age group 60 + years, and 17 (26%) focused on childhood cancer survivors. With regards to outcomes, 82% of 23 studies reporting on physical function showed reduced physical function, 74% of 39 studies reporting on cognitive functional outcomes found reduced cognitive function, and 80% of 15 studies reporting on frailty found increasing frailty among cancer survivors treated with chemotherapy over time and/or compared to individuals not treated with chemotherapy. Fourteen studies (22%) evaluated biologic mechanisms and their relationship to aging-related outcomes. Inflammation was consistently associated with worsening physical and cognitive functional outcomes and epigenetic age increases. Further, DNA damage was consistently associated with worse aging-related outcomes.

**Conclusion:**

Chemotherapy is associated with reduced physical function, reduced cognitive function, and an increase in frailty in cancer survivors; these associations were demonstrated in longitudinal and cross-sectional studies. Inflammation and epigenetic age acceleration are associated with worse physical and cognitive function; prospective observational studies with multiple time points are needed to confirm these findings.

**Implications for cancer survivors:**

This scoping review highlights the need for interventions to prevent declines in physical and cognitive function in cancer survivors who have received chemotherapy.

**Supplementary Information:**

The online version contains supplementary material available at 10.1007/s11764-024-01589-0.

## Introduction

The growing population of cancer survivors is a result of advances in cancer screening, diagnosis, treatment, and supportive care [1, 2]. As of January 1, 2022, it was estimated that there were over 18 million Americans with a history of cancer [3]. With improvements in cancer care extending survival, aging-related physical and cognitive functional changes, frailty, and quality of life become even more important to understand and evaluate in cancer survivors [4, 5]. The concept of accelerated aging refers to the process whereby an individual experiences aging-related changes at a faster rate than average [6]. Aging-related functional declines as a consequence of cancer and its treatment are associated with several biologic mechanisms, including DNA damage, epigenetic dysregulation, mitochondrial damage, cellular senescence, oxidative stress, and chronic inflammation [7]. Cancer survivors encounter functional declines typically associated with aging at earlier chronological ages than their cancer-free counterparts [8, 9].

The National Cancer Institute (NCI) organized a think tank in 2018 [10] to review and summarize the state of the science related to measuring and identifying aging phenotypes in cancer survivors. Participants at the “Measuring Aging and Identifying Aging Phenotypes in Cancer Survivors” think tank reviewed a framework proposed by Ahles and Hurria, positing that cancer and/or cancer treatment could lead to an aging trajectory that is shifted (i.e., Accentuated Aging Hypothesis) or an aging trajectory with an increased rate of functional decline (i.e., Accelerated Aging Hypothesis) [11]. Based on available evidence, the 2018 think tank participants hypothesized that chemotherapy may substantially affect aging-related physical and functional outcomes through increased inflammation, persistent DNA damage, decreased telomere length, and other mechanisms [10]. The think tank summary concluded, "More research is needed to better assess the rate of aging and to understand the relationships between markers of biological age and functional outcomes in cancer survivors” [10].

In this scoping review, we provide an update on the scientific evidence generated since the 2018 NCI think tank [10]. We focus on aging-related outcomes (i.e., physical function, cognitive function, and frailty) in cancer survivors after chemotherapy, given the published evidence demonstrating associations between chemotherapy and aging-related biologic mechanisms [10]. We summarize observational studies (i.e., cross-sectional and longitudinal) published after the 2018 NCI think tank that investigate the relationships between chemotherapy and physical functional outcomes, cognitive functional outcomes, and frailty among cancer survivors. We report on the relationships between biologic mechanisms and aging-related outcomes in these studies.

## Methods

This review followed the Preferred Reporting Items for Systematic Reviews and Meta-Analyses extension for Scoping Reviews (PRISMA-ScR) recommendations (Data Supplement) [12]. Essential components of a PRISMA scoping review include a systematic search strategy, clear inclusion criteria, presentation of the study selection processes, and synthesis of key findings of the included studies [12]. We collaborated with a health sciences librarian (JM) to design a comprehensive search of relevant databases to identify literature evaluating physical functional outcomes, cognitive functional outcomes, and frailty among cancer survivors treated with chemotherapy. We searched for articles from the following databases published between August 2018 and July 2023: PubMed, Web of Science, and Embase. The search involved the integration of standardized terms to retrieve studies about cancer survivors, accelerated aging, functional changes, cognitive changes, and frailty. The specific search terms are detailed in the Data Supplement. We also identified additional relevant studies by screening the reference lists of relevant articles (i.e., “snowball” search). The snowball strategy is approved per PRISMA guidelines [13–16].

### Inclusion criteria

Studies were included if they investigated aging-related outcomes (i.e., physical functional outcomes, cognitive functional outcomes, and/or frailty) among cancer survivors treated with chemotherapy. To extend the findings from the 2018 think tank [10], studies needed to (a) evaluate physical function, cognitive function, and/or frailty as outcomes; (b) include patients with cancer (either the whole sample or a subgroup); (c) include individuals who received chemotherapy treatment (either the whole sample or a subgroup); (d) be observational studies (i.e., employ cross-sectional or longitudinal design); and (e) be written in English. Physical functional outcomes included one or more of these categories: 1) physical performance (e.g., objective tests of gait speed, lower extremity performance, physical activity); 2) patient-reported functional status (e.g., self-reported ability to complete daily tasks such as bathing or cooking), 3) and health-related quality of life (HRQOL) with physical functional components. We included studies that evaluated cognitive functional outcomes and frailty with self-reported and/or objective measures outlined in the 2018 think tank [10, 17, 18]. In some cases, manuscripts were identified by our search that included both the aging-related outcomes of interest and biologic measures (e.g., epigenetic markers, inflammatory markers). In addition to synthesizing information on the relationship between chemotherapy and aging-related outcomes, we summarize the relationships between biologic measures and these outcomes.

### Exclusion criteria

The exclusion criteria for this review were as follows: (a) abstract only; (b) languages other than English; (c) review article, interventional trial, and case study; (d) did not include patients with cancer who received chemotherapy; (e) studies that examined biologic measures without linkage to patient-oriented aging-related outcomes; (f) studies that examined the effects of hormonal therapy, radiation, or surgery alone.

### Search strategy/data charting

Two researchers (MM and MA) reviewed the articles independently to check for the inclusion/exclusion criteria by title and abstracts. After downloading or ordering each report’s full text, the two reviewers thoroughly examined eligibility again and, if included, extracted the data (described below). Duplicate articles were excluded. Disagreements between the two reviewers at each step were resolved by consensus after reviewing the full text, or if consensus could not be reached, a third researcher (SM) made the final decision.

The following information was extracted from each article: first author, year of publication, country of the first author, type of study, sample size at baseline, type of cancer, chemotherapy history, age group, assessment time points, description of how aging-related outcome was measured, and primary findings. Whenever possible, we assessed changes in outcomes over time, accelerated aging (if three or more time points and a longitudinal comparison group with similar time-point assessment intervals were included), and if the investigators reported that the results were statistically significant and/or clinically meaningful. The reported sample sizes exclude the number of healthy participants who were not diagnosed with cancer.

## Results

### Identification of relevant studies (Fig. 1)

**Fig. 1:**
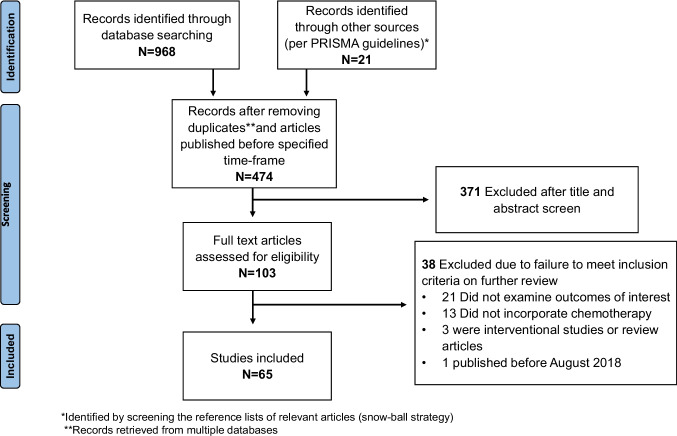
PRISMA flow diagram. This diagram details our search and study selection process applied during the study according to PRISMA checklist

Our search yielded 989 potentially relevant articles (968 articles through the databases searched and 21 articles through the snowball search). After removing duplicates and articles published before the specified timeframe, 474 unique articles remained (Fig. 1). These articles were screened for eligibility, and 371 were determined not to meet the inclusion criteria. The remaining 103 articles underwent full-text assessment, leading to the exclusion of an additional 38 articles. Ultimately, 65 articles that met the inclusion criteria were included. Figure 1 depicts the study selection process.

### Study characteristics (Table 1)

**Table 1:** Characteristics of Included Studies by Age Group^

	Overall (n = 65)	Older age group (60 +) (n = 20)	Childhood group (n = 17)	Adults or no specific age group focus (n = 28)
Cancer type				
Breast	31	15 (48%)	0 (0%)	16 (52%)
Lymphoma/ leukemia/ sarcoma	13	1 (8%)	9 (77%)	2 (15%)
Gastrointestinal	5	1 (20%)	0 (0%)	4 (80%)
Multiple/ others	16	3 (19%)	7 (44%)	6 (38%)
Aging-related outcomes*				
Physical functional outcomes	23	9 (39%)	7 (30%)	7 (30%)
Cognitive functional outcomes	39	10 (26%)	10 (26%)	19 (47%)
Frailty	15	4 (27%)	7 (45%)	4 (27%)
Study design				
Longitudinal	49	15 (31%)	11 (22%)	23 (47%)
Cross-sectional	16	5 (31%)	6 (38%)	5 (31%)
≥ three assessment time points	36	12 (33%)	3 (8%)	19 (53%)

^Age is at cancer diagnosis.

*Percentage may exceed 100% as some studies examined more than one outcome.

Studies were published between August 2018 and July 2023. Most studies were conducted in the United States (n = 50). With respect to publication type, 49 articles were longitudinal studies (Tables 2, 3 and 4), and 16 were cross-sectional studies (Supplemental Table 1). The cancer survivors included in these studies ranged from 18 to 88 years of age. Twenty studies (31%) were limited to the age group 60 + [19–38], while 17 studies (26%) were limited to childhood cancer survivors [39–56]. The time since cancer diagnosis varied widely, ranging from one month to 30 years. The most common types of cancer were breast cancer (46%) [19, 22–24, 26–31, 33–35, 38, 57–72], followed by lymphomas, leukemias, sarcomas (20%) [32, 39–42, 44, 45, 47, 48, 52, 55, 73, 74], gastrointestinal cancers (8%) [37, 54, 75–78] and lung cancer (3%) [79, 80]. Study characteristics, including the age group of patients enrolled and their cancer types, outcomes examined, and number of time points, are summarized in Table 1.

### Aging-related outcomes

Cognition was the most frequently investigated outcome, with 60% of the studies assessing cognitive functional outcomes. Physical functional outcomes were examined in 35% of the studies. Frailty was investigated in 23% of the studies. Eighteen percent of the studies examined more than one outcome (e.g., cognition and frailty).

#### Studies examining cognitive functional outcomes among cancer survivors (n = 39)

Thirty-nine studies (60%) examined cognitive functional outcomes among cancer survivors; 32 were longitudinal in design (Table 2). Twenty-one studies (n = 21/39; 54%) examined cognitive functional outcomes over three or more time points [22, 28, 30, 31, 36, 42, 47–49, 59, 60, 66, 68–70, 73, 75, 77, 78, 81]; of these, 19 (n = 19/21, 90%) included a comparison group, allowing for an assessment of possible accelerated aging [28, 31, 33, 36, 42, 47–49, 59, 66, 68–70, 73, 75, 77, 78, 81]. The most commonly used cognitive measures were Trail Making Test A/B (n = 14/39; 36%) [22–24, 28, 30, 48, 49, 52, 53, 59, 66, 71, 73, 84] and Mini-Mental State Examination (MMSE) (n = 4/39; 10%) [32, 75, 78, 81]. Examples of other measures used to assess cognition include the Cambridge Neuropsychological Test Automated Battery (CANTAB) [59, 73], NIH Toolbox for the Assessment of Neurological and Behavior Function Cognition (NIH-TB) [53], Montreal Cognitive Assessment (MoCA) [77], and Controlled Word Association Test [41, 53, 59]. For self-report measures, the Functional Assessment of Cancer Therapy-Cognition (FACT-Cog) was commonly used (n = 9/39; 23%) [22, 31, 61, 63, 69, 72, 73, 78, 82] followed by Childhood Cancer Survivor Study Neurocognitive Questionnaire (CCSS NCQ; n = 4/39; 10%) [43–45, 47]. Among 19 longitudinal studies examining patients with a parallel comparison group, each with three or more time points, 16 (n = 16/19; 84%) found that cancer survivors receiving chemotherapy developed cognitive decline based on one or more of tests, relative to a control group assessed at similar time intervals, indicating possible accelerated cognitive aging [22, 28, 31, 33, 36, 42, 47–49, 59, 66, 68–70, 73, 75, 77, 78, 81]. Six of these studies (n = 6/16; 37%) reported clinically meaningful differences between survivors and controls [22, 31, 42, 49, 70, 73]. Only three studies (n = 3/19; 16%) found no difference in cognitive functional outcomes pre- to post-chemotherapy or between those who received chemotherapy and the healthy control groups [37, 78, 81]. In one of these studies, the majority of patients with cancer did not receive chemotherapy; when the patients were stratified based on the types of cancer treatment they received, those who received chemotherapy declined slightly faster than other groups [81].

**Table 2:** Longitudinal Studies Examining Cognitive Functional Outcomes Among Cancer Survivors Between August 2018 and July 2023 (*n* = 32)

Author	Country	Cancer Type	Age Group	Sample Size*	Assessment Time	Objective	How cognitive outcomes were measured?	Primary finding	Statistical significance/ Clinically meaningful results
Janelsins[59] 2018	USA	Breast	21 +	N = 580	Pre-chemotherapy, post chemotherapy and 6 months post-chemotherapy	Understand the trajectory and severity of cancer-related cognitive impairment among patients with early breast cancer receiving chemotherapy	Cambridge Neuropsychological Test Automated Battery (CANTAB) Hopkins Verbal Learning and Memory Test–Revised, the Trail Making Test A and B Controlled Oral Word Association (COWA) test. Rey Auditory Verbal Learning Test (RAVLT), digits backward category fluency and backward countingSingle-item self-report assessments. Self-report: participants rated their level of difficulty over 7 days on three single items in specific cognitive domains (remembering things, paying attention, and multitasking) as part of a modified MD Anderson Symptom Inventory	Cancer-related cognitive impairment (memory, attention, executive function) existed for at least 6 months post-chemotherapy compared with noncancer controls	S
Mandel-blatt[22] 2018	USA	Breast	60 +	N = 344	12- and 24-months post-diagnosis	Determine whether older survivors exposed to chemotherapy would have lower neuropsychological domain and self-reported cognitive scores over time than survivors who received hormonal therapy only or controls	Self-reported FACT-cog Declines of 5% to 7%, or 7 to 10 points, were considered clinically meaningful. Objective: Neuropsychological tests that measured attention, processing speed, executive function (APE) and learning and memory	Older survivors exposed to chemotherapy had lower neuropsychological domain and self-reported cognitive scores over time than other groups	S CM
Van der Willik[68] 2018	Nether-lands	Breast	Adults, 50–80 years	N = 166	3 time points Over 8 months	Investigate global cognitive performance, levels of blood cell–based inflammatory markers (granulocyte-to-lymphocyte ratio (GLR), platelet-to-lymphocyte ratio (PLR), and systemic immune-inflammation index (SII)), and their relation in breast cancer survivors who had received post-surgical radiotherapy and chemotherapy on average more than 20 years previously. Examine whether the association between inflammation and cognitive performance were differentially associated between breast cancer survivors and cancer-free women from a population-based sample	Extensive neuropsychological test battery (Mini–Mental State Examination Letter-Digit Substitution Test Word Fluency Test, Stroop Test Purdue Pegboard Test (PPB) (right, left, and both hands) 15-Word Learning Test	Breast cancer survivors had worse cognition than women without a history of cancer. Cancer survivors who had been treated with chemotherapy on average more than 20 years ago have higher blood cell–based inflammatory markers compared with women without a history of cancer. The association between higher levels of inflammatory markers and cognition was statistically significant in cancer survivors but not among non-exposed participants	S S S
Oh^74^ 2019	Korea	Stomach & Colorectal cancer	Mean age 55 years	N = 67	Up to 6 months after chemotherapy (Prechemotherapy, post chemotherapy, and follow up)	Compare changes in cognitive function following chemotherapy between cancer survivors and healthy controls	Self-report: Korean version of Everyday Cognition (Cutoff point of 2 was used for impaired cognitive function) Objective: Korean Mini Mental State Examination (26 points or less cutoff point for decline cognitive function)	Both self-reported and objective cognition declined in chemotherapy group. No differences in cognitive function over time for control group	S
Gregoro-witsch [70] 2019	Netherlands	Breast	Adults	N = 979	Baseline, 3, 6, 12, 18, and 24 months after diagnosis	Evaluate self-reported subjective cognitive function in early-stage breast cancer patients in different age groups up to 2 years after treatment, and to compare outcomes between patients treated with and without chemotherapy. Compare subjective cognitive function scores of patients were compared with cross-sectional scores of an age-matched Dutch reference population including women without a history of breast cancer	Subjective cognitive functioning measured using EORTC QLQ-C30 A decline of 10 points on the EORTC QLQ-C30 cognitive function scale was considered clinically meaningful	Subjective self-reported cognitive functioning of patients treated with chemotherapy, was significantly worse 3, 6, and 12 months compared to non-chemotherapy patients. (Clinically meaningful). The impact of chemotherapy on subjective cognitive function was most pronounced in patients < 55 years, followed by those between 55 and 65 years. Compared to the age-matched reference population, cognitive function of both the chemotherapy and non-chemotherapy patients was lower at all time points	S CM S S
Sales[76] 2019	Brazil	Colorectal cancer (CRC)	Mean age 62 years	N = 580	Before chemotherapy and 12 months after chemotherapy	Evaluate the effects of adjuvant chemotherapy on the cognitive performance of patients with localized CRC compared with a control group	A battery of 13 neuropsychological tests that included verbal and visual memory, attention, visuospatial, and executive function Standardized scores (z-scores) were used to construct a composite score with equal weights for each test	Patients with CRC who received adjuvant 5-fluorouracil with or without oxaliplatin presented with a decline in executive function after 12 months compared with patients with localized disease who had not received chemotherapy. No difference was found in the global composite score, attention, and memory	S NS
Lange[23] 2019	France	Breast	65 +	N = 118	Before adjuvant therapy and after the end of the first adjuvant treatment	Describe objective cognitive changes before to after adjuvant treatment in older adults with early‐stage breast cancer compared with healthy controls	Objective cognitive functioning (episodic memory, working memory, processing speed, and executive functions) was assessed with: Grober and Buschke procedure The Rey Complex Figure Test Wechsler Adult Intelligence Scale‐III the trail making test, (parts A and B) The verbal fluency test. Decline was assessed using Reliable Change Index (RCI); A significant change was found at -1.645 points	51% of patients had no cognitive decline after cancer treatment. Cognitive changes of these patients were not significantly different from changes of the healthy control group	NS
Regier[77] 2019	USA	Oral and GI cancers	40 +	N = 88	6 and 18 months after diagnosis	Examine late cognitive effects in a population of older, male cancer survivors compared to controls	Montreal Cognitive Assessment A cutoff score of < 26 was used to indicate cognitive impairment	48% of cancer patients exhibited cognitive impairment 6 months post-cancer diagnosis, and 40% at 18 months. Cancer survivors were impaired relative to controls on measures of sustained attention, memory, and verbal fluency at 18 months	S
Tonning Olsson[49] 2019	USA	Wilms tumor	Childhood survivors	N = 158	Evaluated annually until they are 18 years of age and at least 10 years post diagnosis	Examine long-term neurocognitive outcomes in survivors of childhood Wilms tumor compared to controls	Wechsler Abbreviated Scale of Intelligence Woodcock Johnson Tests of Achievement III NU California Verbal Learning Test – 2nd edition Trail Making Test Test of Memory and Learning II Controlled Oral Word Association Test To identify clinically relevant impairment, survivors were compared to population norms using one-sample t-tests	Cancer survivors had significantly lowered scores, relative to community controls, in verbal reasoning, word reading, mathematics, sustained attention, long-term verbal memory and verbal fluency	S CM
Yao[63] 2019	USA	Breast	21 +	N = 93	Pre- and post-chemotherapy	Characterize the changes in leukocyte DNA methylome from pre- to post-chemotherapy. Examine significant methylation changes with perceived cognitive impairment	FACT-Cog Decline means any decrease in score	Epigenetic changes (CpG, cg16936953) were associated with cognitive decline in breast cancer patients	S
Alhareeri[60] 2020	USA	Breast	21 +	N = 77	Up to 2 years following chemotherapy at five time-points	Assess the potential relationship between telomere length and development of psychoneurological symptoms	Central Nervous System (CNS) Vital Signs computerized neurocognitive testing system	Chromosomal telomere length was associated with 7 of the 8 cognitive domains evaluated, with the strongest relationship being noted for chromosome 17 and the visual memory domain (shorter telomeres; lower scores)	S
Tonning- Olsson[48] 2020	USA	Soft tissue sarcoma	Childhood survivors Median 33 years	N = 150	Evaluated annually until they are 18 years of age and at least 10 years post diagnosis	Examine long-term neurocognitive outcomes in survivors of childhood soft-tissue sarcoma	Wechsler Abbreviated Scale of Intelligence Wechsler Adult Intelligence scale Woodcock-Johnson Tests of Achievement III Trail Making Test Test of Memory and Learning II Controlled Oral Word Association Test	Compared to controls, survivors demonstrated lower performance on measures of verbal reasoning, mathematics, and long-term memory	S
Bhatt[52] 2021	USA	AML	Survivors of childhood cancer	N = 133	≥ 10 years from initial diagnosis; one assessment	Neurocognitive function was examined among survivors of childhood acute myeloid leukemia compared to healthy controls	Trail Making Test Part A Visual Selective Reminding (new visual learning) Wechsler Digital Span Forward (span) Grooved Pegboard Test Wechsler Symbol Search and Digit Symbol Trail Making Test Part B Controlled Oral Word Association Test Wechsler Digit Span Backward Age-adjusted Z-scores ≤ − 1 and > − 2 SD were considered mildly impaired (grade 1), ≤ − 2 and > − 3 SD moderate (grade 2), and ≤ − 3 SD severe (grade 3)	Survivors were most likely to develop moderate to severe impairments in processing speed and attention when compared to age- and sex-adjusted controls	S
Salerno[69] 2021	USA	Breast	Adults; mean 53 years	N = 580	Pre-chemotherapy, post-chemotherapy, and 6 months post-chemotherapy	Evaluate the association between physical activity and cognitive function	FACT-Cog Delayed Match to Sample, Rapid Visual Processing measures Changes over time of more than 1/2 SD of the FACT-Cog measured at baseline were clinically meaningful	Patients demonstrated comparable scores as controls on Delayed Match to Sample and Rapid Visual Processing measures. Patients reported more decline than controls on the FACT-Cog	NS S
Williams[42] 2021	USA	Lymphoma, leukemia, sarcoma, CNS, Wilms	Childhood survivors 18–45 years	N = 845	Initial visit ≥ 10 years from diagnosis and single visit 5 years after study entry	Characterize the association between frailty and neurocognitive impairment and the prospective association between frailty and neurocognitive decline over a period of approximately 5 years in young-adult survivors of childhood cancer	Neurocognitive tests of intelligence and academics, attention, processing speed, memory, and executive function Decline was measured by assessing the change in age-adjusted neurocognitive *Z* scores (continuous outcome) and compared between groups	Frail survivors declined an average of 0.54 standard deviations in short-term verbal recall, whereas non-frail survivors did not decline. Frail survivors declined more than non-frail survivors on visual-motor processing speed, cognitive flexibility, and verbal fluency. Prefrail and frail survivors experienced greater declines in focused attention compared with non-frail survivors	S CM S S
Van der Willik[81] 2021	Netherlands	Multiple cancers	55 +	N = 2403	Every 3–6 years	Assess cognitive trajectories of non- central nervous system cancer patients before and after cancer diagnosis in a population-based setting	Mini-Mental State Examination (MMSE) Neuropsychological battery including the Letter-Digit Substitution Test, Word Fluency Test, and Stroop Test	Cognitive function did not change differently over time between individuals who were diagnosed with cancer and controls	NS
Williams[46] 2021	USA	Multiple cancers	Childhood cancer survivors	N = 2,859	≥ 10 years from diagnosis. One timepoint	Examine whether children who experience central nervous system injury are at higher risk for neurocognitive impairment associated with subsequent late onset chronic health conditions	Comprehensive neurocognitive assessment that included tests of intelligence, attention, processing speed, memory, and executive function Neurocognitive impairment was defined as a Z-score below the 10th percentile	CNS-treated survivors performed worse than non-CNS-treated survivors on all neurocognitive tests	S
Stefanski[47] 2021	USA	AML	Childhood cancer survivors; < 21 years at diagnosis	N = 482	≥ 5 years from diagnosis (baseline and 4 follow up surveys)	Evaluate neurocognitive and psychosocial outcomes in long-term AML survivors treated with bone marrow transplantation (BMT) or intensive chemotherapy without BMT compared to siblings	Childhood Cancer Survivor Study Neurocognitive Questionnaire (CCSS NCQ) Impairment means presence of at least 2 elevated CCSS-NCQ scales	AML survivors were more likely than siblings to report impairment in overall neurocognitive outcomes	S
De- Rosa[82] 2021	Italy	Gynecological cancer	Adults	N = 73	Before starting treatment and 6 months after the end of treatment	Evaluate the perception of cognitive decline in patients undergoing surgical and / or medical therapy	FACT-Cog	A significant reduction in perceived cognitive impairments was demonstrated at 6 months	S
Van- Dyk[30] 2021	USA	Breast	60 +	N = 427	Every year for 2 years	Evaluate of the role of *APOE* ε2 in longitudinal cognitive function among older breast cancer survivors and a matched control group	Neuropsychological assessment measured attention, processing speed, executive function, and learning and memory: APE Trail making A and B Digital forward and backward Controlled Oral Word Association Task (COWA) Logical memory	There was an interaction (borderline significance) between the chemotherapy group (versus control) and genotype for attention, processing speed, and executive functioning domain scores. There was no effect of ε2 on learning and memory domain scores	NS
Wang[36] 2021	USA	Multiple cancers	65 +	N = 1564	Biannually for 15 years	Examine whether cancer history accelerates older adults’ rates of cognitive decline over time; examine whether chemotherapy increases older cancer survivors’ rates of cognitive decline over time	Composite score of mental status and episodic memory using reduced version of Telephone Interview for Cognitive Status (TIC)	Middle-old adults (aged 75–84) with a cancer history had significantly reduced rates of cognitive decline over time, including the global measure of cognitive functioning, mental status, and episodic memory compared to their counterparts without a cancer history	S
Janelsins[73] 2022	USA	lymphoma	21 +	N = 248	Pre-chemotherapy, post-chemotherapy, and 6 months post-chemotherapy	Assess changes in memory, attention, and executive function in patients with lymphoma from pre- to post-chemotherapy and to 6 months post-chemotherapy compared with controls	CANTAB Hopkins Verbal Learning and Memory Test–Revised, the Trail Making Test A and B Controlled Oral Word Association (COWA) test. Rey Auditory Verbal Learning Test (RAVLT), digits backward category fluency and backward counting Single-item self-report assessments. FACT-Cog 1/2 SD was used as a cutoff for a minimal clinically important difference	On FACT-Cog, Patients reported more cognitive problems from pre- to post-chemotherapy and from pre-chemotherapy to 6 months follow-up, compared with controls. Patients with lymphoma performed statistically significantly less well on tests of verbal memory and delayed recall, attention and executive function, and telephone-based category fluency	S CM S
Ahles[28] 2022	USA	Breast	60 +	N = 328	Assessments occurred at enrollment and at 8, 16, and 24-month follow-ups	Examine the trajectory of cognitive aging in older, disease-free, long-term breast cancer survivors who were assessed 5–15 years after diagnosis compared to controls. Examine the relationship between frailty and cognitive function in older, long-term breast cancer survivors	Cognitive Reserve: Wide Range Achievement Test 4 (WRAT4) Language: Category Fluency; Boston Naming Test, Attention, Processing Speed, Executive Function: Digit Symbol; Trail Making A and B; DKEFS Color-Word Naming; NAB Digits Forward and Backward; NAB Driving Scenes Learning and Memory: NAB List Learning: Trial 1, Semantic Clustering, List A Immediate, List A Delayed, Long Delay, List B Immediate, New Recognition Index; Logical Memory Part 1 and 2 (WMS-R)	Cancer survivors scored significantly lower on the Learning and Memory compared to controls. Increasing frailty scores were associated with worse cognitive performance across all domains	S S
Ahles[33] 2022	USA	Breast	60 +	N = 328	Assessments occurred at enrollment and at 8, 16, and 24-month follow-ups	Determine whether older breast cancer survivors score lower on neuropsychological tests compared to matched non-cancer controls	Cognitive Reserve: Wide Range Achievement Test 4 (WRAT4) Language: Category Fluency; Boston Naming Test, Attention, Processing Speed, Executive Function: Digit Symbol; Trail Making A and B; DKEFS Color-Word Naming; NAB Digits Forward and Backward; NAB Driving Scenes Learning and Memory: NAB List Learning: Trial 1, Semantic Clustering, List A Immediate, List A Delayed, Long Delay, List B Immediate, New Recognition Index; Logical Memory Part 1 and 2	Breast cancer survivors scored significantly lower on all domains of cognitive function	S
Vardy[78] 2022	Australia/ Canada	CRC	Adults 56–83 years	N = 25	Up to 12 years after diagnosis; assessments conducted after surgery and prior to any chemotherapy; subsequent assessments were 6, 12 and 24 months later	Evaluate cognitive function in CRC survivors 6–12 years after diagnosis compared with healthy controls	MMSE WRAT 3 Reading test A neuropsychological test battery evaluated four cognitive domains: working memory and attention; processing speed; verbal learning and memory; visual learning and memory. Cognitive impairment was defined as a global deficit score of > 0.5 or > 1.5 SD below the normative mean on > 2 tests or > 2 SD on one test. FACT-Cog (A clinically meaningful functional deficit score was defined as ≥ 0.52 points)	There were no significant differences in cognitive scores or proportion with cognitive impairment between survivors and controls	NS
Chipeeva[41] 2022	Russia	lymphoma, leukemia	Childhood cancer survivors: 6–17 years	N = 504	Not reported	Assess the individual differences in cognitive ability and fine motor skills of pediatric tumor survivors and the age-matched healthy controls	Short-term and working memory: Wechsler Memory Scale, Third Edition Digit Span test. Visuospatial constructional ability: Rey–Osterrieth Complex Figure Verbal fluency: Controlled Oral Word Association Task (COWA) Fine motor skills, eye–hand coordination, and motor speed: The Grooved Pegboard	Cancer survivors scored significantly worse in fine motor skill, verbal fluency, and motor tests compared with the control group	S
Fowler[37] 2022	USA	GI cancers	60 +	N = 218	Baseline and one 3–6-month follow-up	Evaluate early longitudinal cognitive complaints and predictors among older adults with cancer	PROMIS short form—cognitive function-4	52% had stable cognition baseline to follow-up (follow-up *t*-score ± 5 points of baseline), 20% improved (≥ 5 increase), and 27% declined (≥ 5 decrease). After adjustment, there were no significant baseline predictors of follow-up cognitive *t*-score	NS
Belcher[66] 2022	USA	Breast	50 +	N = 519	Pre-chemotherapy, post-chemotherapy, and 6 months post-chemotherapy	Evaluate serum cytokine in patients with breast cancer before and after chemotherapy compared with controls; Assess relationships of cytokine and receptor levels with tests of cognitive function	Attention and processing speed were measured by Rapid Visual Processing (RVP) Backward Counting (BCT) Trail Making-A (TMT-A) tests	sTNFRI and sTNFRII increased over time in patients relative to controls. Higher IL-8 associated with worse BCT. Higher IL-4 and IL-10 associated with better TMT-A. Post chemotherapy, higher IL-8, sTNFRII associated with worse BCT	S S S S
Ahles[27] 2023	USA	breast	60 +	N = 220	5–15 years post diagnosis At enrollment and at 8-, 16-, and 24-month follow-ups	Examine whether cognitive function in older, long-term breast cancer survivors is both a direct effect of cancer and cancer treatments and an indirect effect mediated by deficit accumulation	Neuropsychological battery	Cognitive performance was mediated by deficit accumulation for all domains	S
Carroll[31] 2023	USA +	Breast	60 +	N = 400	Baseline and at annual visits up to 60 months	Examine longitudinal relationships between levels of C-reactive protein (CRP) and cognition in older breast cancer survivors and noncancer controls	FACT-Cog A clinically significant decline was a decrease of 7–10 points	Survivors had significantly higher adjusted mean CRP than controls at baseline and 12-, 24-, and 60-month visits. Higher adjusted CRP predicted lower participant-reported cognition on subsequent visits among survivors, but not controls	S S CM
Phillips[43] 2023	USA	Multiple	Childhood cancer survivors: < 21 years, survived at least 5 years after diagnosis	N = 2375	Up to 35 years after diagnosis. Assessment at baseline and follow-up	Determine whether aging adult childhood cancer survivors report more new-onset neurocognitive impairments compared with their siblings and to identify risk factors associated with such impairments	CCSS NQS A binary outcome of impairment in each domain was defined as a score in the worst 10% of the CCSS sibling cohort based on the distribution of that domain score (mean and SD) of all siblings tested at each survey	New-onset memory impairment emerged more often in cancer survivors compared to their siblings. The increased risk was associated with cancer treatment, modifiable health behaviors, and chronic health conditions	S
Kedan- Lottick[44] 2022	USA	Osteosarcoma Ewing sarcoma	Childhood cancer survivors < 21 years at diagnosis	N = 960	5 years post- diagnosis; 2 timepoints	Evaluate associations between treatment exposures, chronic health conditions, and patient-reported neurocognitive outcomes in survivors of childhood cancers	CCSS NQS	Survivors, vs. siblings, reported higher prevalence of difficulties with task efficiency and emotional regulation. Survivors are at increased risk for reporting neurocognitive difficulties, which are associated with employment status and appear related to chronic health conditions that develop over time	S

Abbreviations: *CANTAB* Cambridge neuropsychological test automated battery, *chemo* chemotherapy, *CCSS NCS* childhood cancer survivor study neurocognitive questionnaire, *CM* clinically meaningful, *FACT* functional assessment of cancer therapy, *MMSE* mini-mental state examination, *NS* non-statistically significant, *S* statistically significant, *SD* standard deviation

* The sample sizes depicted included the number of participants who had a diagnosis of cancer.

The majority of research on cancer-related cognitive function has focused on adults with breast cancer. In 580 patients with breast cancer aged 21 + years, Janelsins et al. found evidence of chemotherapy-related cognitive impairment (CRCI) in multiple domains for at least six months post-chemotherapy, with a difference noted for visual memory compared to controls [59]. Using a variety of patient-reported and objective measures, several studies have demonstrated cognitive functional declines in older patients with breast cancer receiving chemotherapy [22, 31, 70]. For example, Mandelblatt et al. reported impaired attention and reduced processing speed and executive function for up to two years after chemotherapy in 344 patients aged 60 + with breast cancer compared to non-cancer controls [22]; these data are suggestive of accelerated cognitive aging.

Multiple studies have investigated cognitive functional outcomes among childhood cancer survivors. In a study of pediatric cancer survivors aged 6–17 years old by Chipeeva et al., survivors (n = 504) scored significantly worse on measures of memory, visuospatial processing, and verbal fluency than those without a history of cancer [41]. In two studies by Olsson et al., including childhood cancer survivors diagnosed with Wilms tumor and soft tissue sarcoma, survivors had lower scores than community controls in verbal reasoning, word reading, mathematics, sustained attention, long-term verbal memory, and verbal fluency [48, 49]. In a cross-sectional analysis of survivors of Hodgkin’s lymphoma, Williams et al. demonstrated that survivors, compared with their siblings, exhibited impairment in neurocognitive function and were more likely to be unemployed and have a lower income [45]. In a large study by Phillips et al. of childhood cancer survivors (n = 2375) assessed up to 35 years post-cancer diagnosis, new-onset memory impairment emerged more often in cancer survivors than in their siblings [85]. The increased risk was associated with cancer treatment, modifiable health behaviors, and chronic health conditions. Similarly, in a cohort study including 960 childhood cancer survivors aged < 21 years at diagnosis, Kedan-Lottick et al. showed that survivors of childhood osteosarcoma and Ewing sarcoma were at increased risk for reporting neurocognitive difficulties, which were associated with employment status and chronic health conditions that developed over time [44].

#### Studies examining physical functional outcomes among cancer survivors (n = 23)

Twenty-three studies (35%) examined physical functional outcomes among cancer survivors; 17 were longitudinal in design (Table 3). Sixteen studies (n = 16/23; 69%) examined physical functional outcomes over three or more time points [19–21, 26, 34, 35, 47–49, 57, 58, 69, 78–80, 83]; eight (n = 8/23; 35%) included a comparison group allowing for an assessment of possible accelerated aging in this domain [34, 47–49, 58, 69, 78, 83]. Across studies, a broad spectrum of measures evaluating various physical functional outcomes were used. Most studies (n = 20/23; 87%) used patient-reported outcomes (PRO) to assess physical function among cancer survivors. The PRO most commonly utilized was the Medical Outcomes Study Questionnaire Short Form -36 (SF-36) (n = 12/23 studies; 52%) [29, 40, 45, 47–49, 56–58, 79, 83]. Among three studies (n = 3/23; 13%)[29, 52, 69] that examined objective physical performance only, the most common measure was the Short Physical Performance Battery (SPPB) test. Overall, 19/23 (82%) of studies documented an association of chemotherapy with reduced physical function over time in longitudinal studies and/or differences in physical functional outcomes between those participants who received chemotherapy and those who did not [19–21, 26, 34, 35, 40, 45, 48, 57, 58, 79, 80, 83]. The six cross-sectional studies demonstrated physical function differences among cancer survivors receiving chemotherapy compared to participants who did not [32, 38, 40, 45, 56, 86]. Among the 16 longitudinal studies with at least three assessment points, 81% (n = 13/16) demonstrated statistically significant changes over time [19–21, 26, 34, 38, 47, 48, 57, 58, 69, 80, 83]. Among the eight longitudinal studies with a comparison group, 75% (n = 6/8) showed greater physical functional declines over time in cancer survivors than in controls, indicating evidence of accelerated aging among cancer survivors [34, 47, 48, 58, 69, 83].

**Table 3:** Longitudinal Studies Examining Physical Functional Outcomes Among Cancer Survivors between August 2018 and July 2023 (n = 17)

Author	Country	Cancer type	Age Group	Sample Size*	Assessment Time	Objective	How functional outcomes were measured?	Primary finding	Statistical significance/ Clinically meaningful results
Wong[20] 2018	USA	Multiple	65 +	N = 363	Prior to chemotherapy administration; 1 week after chemotherapy administration;2 weeks after chemotherapy administration	Determine demographic, clinical, and symptom characteristics associated with baseline function as well as trajectories of physical function over two cycles of chemotherapy	Short Form (SF)-12 Physical Component Summary (PCS) score	PCS scores decreased by 0.21 points at each subsequent assessment. Morning fatigue and lower baseline PCS associated with decreases in physical function over time	S S
Hurria[19] 2019	USA	Breast	65 +	N = 256	Pre chemotherapy; End of chemotherapy; 1 year after chemotherapy initiation	Describe self-reported changes in physical function in older adults receiving adjuvant chemotherapy during the first year after treatment	Physical function subscale of the European Organization for Research Treatment of Cancer Quality of Life Questionnaire Decline was defined by ≥ 10-point decrease from pre-chemotherapy to end-chemotherapy (clinically meaningful)	In 42% of participants who had physical function decline from before to the end of chemotherapy, 47% recovered by 12 months. One-third experienced functional decline from before chemotherapy to 12 months later. Baseline fatigue was associated with decline in physical function from pre-chemotherapy to end of chemotherapy	CM S
Presley[21] 2019	USA	Multiple	70 +	N = 170	Monthly for 1 year after cancer diagnosis	Characterize functional trajectories in the year after a new cancer diagnosis among older adults	Disability defined by 13 items form Activities of Daily Living (ADL) and Instrumental ADL (IADL) scales. Worsening disability post-diagnosis was defined as an increased score on the 13-item disability scale during the 12-follow-up period, relative to pre-diagnosis	Most participants (94%) with severe disability pre-diagnosis had severe disability post-diagnosis. 40% of participants with a mild or moderate disability pre-diagnosis transitioned to a worse functional trajectory post-diagnosis. Risk factors independently associated with worsening disability post-diagnosis included moderate or severe disability pre-diagnosis, poor physical capability, and incurable stage	S
Tonning -Olsson[49] 2019	USA	Wilms tumor	Childhood cancer survivors	N = 158	Evaluated annually until they are 18 years of age and at least 10 years post diagnosis	Examine long-term functional outcomes in survivors of childhood Wilms tumor	General health index SF-36 (physical function is subscale) Impairment means > 1 SD below the mean. To identify clinically relevant impairment, survivors were compared to population norms using one-sample t-tests	No difference between survivors and controls in the physical function index. Survivors had lower scores (0.26 mean difference) on the general health index	NS S
Avis[57] 2020	USA	Breast	42 to 52 years old	N = 141	At baseline and approximately annually up to 10 years post diagnosis	Compare health-related quality of life (HRQL) from diagnosis to 10 years post diagnosis among breast cancer survivors and women without cancer over the same period	HRQL using SF-36 Decline means changes of 2 points or more	Breast cancer survivors had significantly lower HRQL compared with controls at diagnosis and 1-year post diagnosis. By 2 years, breast cancer survivors and controls did not have significantly different scores	S NS
Michael[58] 2020	USA	Breast	50 + (Postmenopausal)	N = 1636	Up to 12 years post diagnosis. At baseline and at years 3, 11, and 12	Evaluate the effect of breast cancer on change in physical function compared with women without breast cancer	Physical function using 10 items from the RAND Short Form 36 scale, a well-validated measure of self-reported SF-36 Clinically meaningful decline means ≥ 0.8 points	Women with breast cancer experienced clinically meaningful greater physical function decline 2.24 points compared with women without breast cancer. Breast cancer effect on physical function was greater among women in older age groups	S CM S
Kobayashi[35] 2020	USA	Breast	60 +	N = 397	Up to 2 years after diagnosis; baseline, 12, and 24-months post-baseline	Examine the relationships between cognition prior to systemic therapy and subsequent well-being (i.e., functional outcomes) over 24 months in older breast cancer survivors	Global wellbeing using FACT-G Cognition using FACT-Cog Minimum clinically important differences mean ≥ 3.6 points	Self-reported cognitive impairment using FACT-Cog was associated with lower global well-being over the first 2 years of survivorship	S CM
Tonning-Olsson[48] 2020	USA	Soft tissue sarcoma	Childhood cancer survivors Median age 33 years	N = 150	Evaluated annually until they are 18 years of age and at least 10 years post diagnosis	Examine long-term functional outcomes in survivors of childhood soft-tissue sarcoma compared to a control group	HRQL using SF-36 (physical function is subscale) Impairment means > 1 SD below the mean	Survivors had significantly lower HRQOL and physical function on all measures compared to the control group, and nearly all subscales were lower than normative data	S
Medysky[79] 2021	USA	Lung	18 +	N = 72	Baseline (within 6 months of diagnosis) and at 3, 6, 9, and 12 months of chemotherapy	Identify inter-individual differences in the pattern and rate of change in self-reported functioning in lung cancer survivors and examine whether and how symptoms are related to physical functioning over time	Physical function using SF-36 Scores were transformed to 0 to 100, with 50 indicating the population average and high scores indicating better function	Average physical functioning did not decrease over time. Fatigue, assessed over 1 year, was predictor of physical functioning changes over time	NS S
Stefanski[47] 2021	USA	Acute Myelogenous Leukemia (AML)	Childhood survivors. < 21 years old at diagnosis	N = 482	≥ 5 years from diagnosis (baseline and 4 follow up surveys)	Evaluate neurocognitive and QoL (function) outcomes in long-term AML survivors treated with bone marrow transplantation (BMT) or intensive chemotherapy without BMT compared to siblings	Physical function using SF-36 SF-36 scores of no more than 1 SD below the normative mean were classified as impaired	AML survivors were more likely than siblings to report impairment in physical function	S
Bhatt[52] 2021	USA	AML	Childhood cancer survivors	N = 133	≥ 10 years from initial diagnosis; baseline and one assessment later were conducted	Compare physical function among survivors of childhood AML to healthy controls	Exercise physiology in six domains: aerobic function (Six-Minute Walk) Physiologic Cost Index), mobility (Timed Up and Go, strength (hand grip strength, knee extension at 60°/second, endurance (knee extension at 300°/second), flexibility (passive dorsiflexion, active dorsiflexion, Sit and Reach Test), and balance (Sensory Organization Test, vestibular score) Wechsler Digit Span Backward Impairment in survivors was defined as > 1.5 SD below the age-, gender-matched Z-score for controls	Survivors had a higher prevalence of physical function impairment compared to controls across all domains, except balance	S
Salerno[69] 2021	USA	Breast	Adults; mean age 53 years	N = 580	Pre-chemotherapy, post-chemotherapy, and 6 months post-chemotherapy	Evaluate patterns of physical activity before, during, and after chemotherapy in patients with breast cancer compared to controls	Aerobics Center Longitudinal Study Physical Activity measure	Patients had declines in physical activity from pre-chemotherapy to post-chemotherapy compared with controls. From post-chemotherapy to 6 months post-chemotherapy, patients reported significantly increased physical activity across all outcomes compared with controls	S S
Presley[80] 2022	USA	Non-small cell lung cancer (NSCLC)	18 +	N = 207	Monthly for 8 months	Evaluate whether patients with advanced NSCLC experience functional disability or have resilience. Identify characteristics associated with functional disability	Function using EuroQol 5 domains (EQ-5D-5L) survey. A 1-point increase in functional status score (increase in disability) was considered a meaningful decline in function, representing a 0.5 SD change on the EQ-5D-5L	Three distinct functional trajectory groups were identified: none/mild (38%), moderate (48%), and severe disability (14%). At month 8 relative to baseline, 46% of the participants were classified as resilient and 5% experienced functional decline. Poor baseline performance status, worse dyspnea and pain, and higher anxiety scores were associated with severe disability	S
Vardy[78] 2022	Australia/ Canada	Colorectal cancer	56–83 years	N = 25	Up to 12 years post diagnosis; Assessments at baseline, 6, 12 and 24 months	Evaluate physical function 6–12 years after cancer diagnosis compared to non-cancer controls	ADL and IADL	There were no differences in functional tests between groups	NS
Rentscher[34] 2023	USA	Breast	60 +	N = 89	Up to 5 years post diagnosis, 24 to 36 and 60 months after enrollment	Examine whether older breast cancer survivors showed greater epigenetic aging (using these measures: (Horvath, Extrinsic Epigenetic Age, PhenoAge, Grim Age, Dunedin Pace of Aging) than controls and whether epigenetic aging related to functional outcomes	Physical function using Medical Outcomes Study Short Form-12	Older breast cancer survivors, particularly those exposed to chemotherapy, showed greater epigenetic aging. An older epigenetic age was associated with worse physical function. Black survivors showed accelerated aging over time relative to non-Hispanic White survivors	S S S
Cespede- Feliciano[83] 2023	USA	Multiple cancers	50 +	N = 9203	At enrollment, 1-year, 3-year, 6-year and 9-year follow-up	Examine trajectories of physical function before and after cancer diagnosis among older survivors and cancer-free controls	Physical function using 10-item RAND-36 scale (higher scores indicating superior	Survivors of cancer experienced declines in physical function (1–2 points per year) that accelerated from pre-diagnosis to post diagnosis at a rate faster than among age-matched controls. Colorectal cancer survivors had physical function similar to age-matched controls by 5 years after diagnosis, while other cancer types had significant deficits in physical function even 5 years after cancer diagnosis	S
Lemij[26] 2023	Netherlands	Breast	70 +	N = 239	At baseline, 3, 9, 15, 27, and 60 months after treatment	Assess changes in physical function in the first 5 years after breast cancer diagnosis in a cohort of older patients and to identify factors associated with physical decline	ADL IADL No clinically significant cutoff was determined	Patients with dependencies in ADL/IADL at baseline remained less active over the first 5 years after breast cancer diagnosis. Geriatric characteristics (age, comorbidities, BMI), depression and loneliness were associated with longitudinal change in physical function. A better quality of life was associated with preservation of function	S S S

Abbreviations: *ADL* activities of daily living, *chemo* chemotherapy, *CM* clinically meaningful, *IADL* instrumental activities of daily living, *FACT* functional assessment of cancer therapy, *EQ-5D-5L* EuroQol 5 domains, *FACT* functional assessment of cancer therapy, *HL* hodgkin lymphoma, *SF-36* short form health survey, *SPPB* short physical performance battery, *NS* non-statistically significant, *S* statistically significant, *SD* standard deviation

* The sample sizes depicted included the number of participants who had a diagnosis of cancer.

Twelve studies (n = 12/23, 52%) examined long-term changes (≥ 5 years) since diagnosis [26, 34, 40, 45, 47–49, 52, 57, 58, 78, 83]. Five studies examined physical functional outcomes for up to one year [19–21, 79, 80]. As examples, Medysky et al. and Presley et al. found that patients with lung cancer aged 18 + years old who had a high level of symptom burden were more likely than those who had a lower symptom burden to experience physical functional declines as measured by HRQOL measures over one year after diagnosis [21, 79].

The majority of longitudinal studies (n = 12/16; 75% of studies that include three or more time points) provided data suggestive of accelerated aging among cancer survivors compared to non-cancer controls. For example, Stefanski et al. found that acute myeloid leukemia survivors treated with intensive chemotherapy were more likely than their siblings to report impairment in physical function as measured by SF-36 scores [47]. In another study of 9203 patients with various cancer types, Cespedes-Feliciano et al. found that cancer survivors aged 50 + years old experienced accelerated declines in physical function post-diagnosis compared to controls [83].

Studies conducted in older patients found physical function declines were common after chemotherapy [19, 29, 34, 57, 58]. Hurria et al. found that of 42% of 256 patients with breast cancer who experienced physical function declines on an HRQOL measure at the end of chemotherapy, only 47% recovered by 12 months [19]. Winters-Stone et al., Avis et al., Micheal et al., and Rentscher et al. found that older survivors of breast cancer were more likely to experience physical functional declines than those without breast cancer [29, 34, 57, 58].

#### Studies examining frailty outcomes among cancer survivors (n = 15)

Among 15 studies (n = 15/65; 23%) that assessed frailty in patients receiving chemotherapy, eight (n = 8/15; 53%) were longitudinal in design (Table 4) [25, 28, 42, 50, 54, 61, 65, 67]. Only six studies (n = 6/15; 40%) examined frailty over three or more time points [28, 42, 54, 61, 65, 67]; five (n = 5/6; 83%) of these studies included a comparison group [28, 42, 54, 61, 65, 67]. Ten studies (n = 10/15; 67%) used the Fried frailty phenotype index (in its original or modified version) [39, 40, 42, 50, 51, 55, 56, 61, 65, 67], while five studies (n = 5/15; 33%) utilized the deficit accumulation index (DAI) [24, 27, 28, 54, 62]. Seven studies (n = 7/15; 47%) examined frailty in a cohort of childhood cancer survivors [39, 40, 42, 50, 51, 54–56], while four studies (n = 4/15; 27%) examined frailty among older adults [24, 27, 28, 62]. All five longitudinal studies that included a comparison group found that cancer survivors experienced increased frailty compared to non-cancer controls [28, 42, 54, 61, 65, 67]. Williams et al. found that childhood cancer survivors aged 18 + years (n = 400) had increased frailty as measured by DAI compared with controls [54]. Another study by Magnuson et al. found that among breast cancer survivors aged 50 + years old, longitudinal declines in FACT-Cog and objective measures of attention and memory were associated with increased frailty during chemotherapy and for up to six months post-chemotherapy compared with controls [61].

**Table 4:** Longitudinal Studies Examining the Change in Frailty Among Cancer Survivors between August 2018 and July 2023 (n = 8)

Author and year	Country	Cancer	Age Group	Sample Size*	Assessment Time	Objective	How frailty was assessed	Results	Statistical Significance/Clinically meaningful results
Magnuson[61] 2019	USA	Breast	50 +	N = 376	Pre-chemotherapy, post-chemotherapy, 6-months post-chemotherapy	Evaluate relationships between frailty and cognition longitudinally in adults 50 years and older with breast cancer receiving chemotherapy	Modified Fried criteria (worse frailty means any increase in score) (i.e., CANTAB and FACT-Cog were used for cognition assessment)	Longitudinal decline in FACT-Cog between pre-chemotherapy and post-chemotherapy and between pre-chemotherapy and 6 months was associated with increased frailty score in patients compared to controls.	S
Hayek[50] 2020	USA	Multiple cancers	Childhood cancer survivors	N = 10,899	Assessment at 5 or more years post diagnosis and another follow up assessment.	Estimate the prevalence of frailty among childhood cancer survivors and to determine the direct and indirect effects of treatment exposures, lifestyle factors, and severe, disabling, and life-threatening chronic condition on frailty	Modified Fried criteria. Participants endorsing ≥ 3 criteria were considered frail	The overall prevalence of frailty among survivors was 3 times higher compared with siblings. Survivors of CNS tumors and bone tumors had the highest prevalence of frailty. Survivors exposed to cranial radiation, abdominal radiation > 40 Gray, cisplatin ≥ 600 mg/m^2^, amputation, or lung surgery had increased risk for frailty	NR S
Gilmore[65] 2020	USA	Breast	Adult 50 +	N = 144	Pre chemotherapy, post chemotherapy, and 6 months later	Determine if pre-chemotherapy inflammation is predictive of frailty after chemotherapy	Frailty assessed using a modified Fried's score	Post- versus pre-chemotherapy, a higher percentage of patients reported increased frailty compared to controls. Patients with pre-chemo serum levels of IL-6, sTNFRII, and sTNFRII above the median were more frail after chemotherapy than those with levels below the median	S
Williams[42] 2021	USA	Lymphoma, leukemia, sarcoma, CNS, Wilms	Childhood cancer survivors	N = 845	≥ 10 years from diagnosis	Characterize the prospective association between frailty and neurocognitive decline over a period of approximately 5 years	Frailty: Fried criteria Participants endorsing ≥ 3 criteria were considered frail. Cognition: Neurocognitive: tests of intelligence and academics, attention, processing speed, memory, and executive function Decline was measured by assessing the change in age-in neurocognitive *Z* scores (continuous outcome)	Compared to non-frail survivors, frail survivors declined in short-term verbal recall, whereas non-frail survivors did not decline. Frail survivors declined more than non-frail survivors on visual-motor processing speed, cognitive, and verbal fluency. Prefrail and frail survivors experienced greater declines in focused attention	S S
Gilmore[67] 2021	USA	Breast cancer	Adult 50 + years	N = 586	Pre chemotherapy, post chemotherapy, and 6 months later	Investigated whether pre-chemotherapy levels of cellular markers of inflammation as well as their change with chemotherapy were associated with post-chemotherapy frailty and frailty that persists up to 6 months after the completion of chemotherapy	Frailty assessed using a modified Fried's score	In post-chemotherapy compared to pre-chemotherapy, patients reported that they had increased frailty compared to controls. Six months after the completion of chemotherapy, patients were less active. All other components of frailty returned to pre-chemotherapy levels 6 months after the completion of their chemotherapy regimen	S
Ahles[28] 2022	USA	Breast cancer	60 + years	N = 328	Assessments occurred at enrollment and at 8, 16, and 24-month follow-ups	Examine the relationship between cognitive function and frailty in older, long-term breast cancer survivors	DAI Continuous DAI scores were used to classify participants as robust (DAI < 0.2), pre-frail (0.2 ≤ DAI < 0.35), or frail (DAI ≥ 0.35) Change in DAI as a categorical variable is clinically meaningful	Survivors had significantly higher DAI scores compared to controls. Increasing frailty scores were associated with worse cognitive performance across all domains	S CM S
Ji[25] 2023	USA	Breast cancer	65 + years	N = 348	Before and after chemotherapy	Evaluate the association between pre-chemo CARG-BC score and decline in frailty, from robust to prefrail or frail health, after chemotherapy	DAI Continuous DAI scores were used to classify participants as robust (DAI < 0.2), pre-frail (0.2 ≤ DAI < 0.35), or frail (DAI ≥ 0.35) Change in DAI as a categorical variable is clinically meaningful	Women with intermediate or high CARG-BC scores had greater odds of decline in frailty compared with women with low scores	S CM
Williams[54] 2023	USA	Lymphoma, leukemia, sarcoma, CNS, Wilms	Childhood survivors 18 +	N = 400	5 or more years post-diagnosis; multiple assessments	Examine premature aging as an accumulation of deficits in pediatric cancer survivors compared with controls	DAI Continuous DAI scores were used to classify participants as robust (DAI < 0.2), pre-frail (0.2 ≤ DAI < 0.35), or frail (DAI ≥ 0.35) Change in DAI as a categorical variable is clinically meaningful	Survivors had a statistically significant and clinically meaningful higher mean DAI after adjustment for age, sex, and race and ethnicity compared with controls	S CM

Abbreviations: *chemo* chemotherapy, *CM* clinically meaningful, *CNS* central nervous system, *DAI* deficit accumulation index, *FACT* functional assessment of cancer therapy, *NS* non-statistically significant, *S* statistically significant, *SD* standard deviation

* The sample sizes depicted included the number of participants who had a diagnosis of cancer.

#### Studies examining biologic measures in relation to physical functional outcomes, cognitive functional outcomes, or frailty (Supplementary Table 2)

Overall, 14 studies (22%) identified in our search examined the relationship between biologic measures and aging-related outcomes among cancer survivors; four of these studies (n = 4/14; 29%) had an endpoint of frailty [55, 56, 65, 66], two (n = 2/14; 14%) had an endpoint of physical function [34, 56], and nine had an endpoint of cognitive function (9/14, 64%) [30, 31, 33, 60, 63, 64, 66, 68, 71]. Two of these studies (n = 2/14; 14%) were conducted with childhood cancer survivors [55, 56], while four studies (n = 4/14; 29%) were restricted to older cancer survivors [30, 31, 33, 34]. Most (n = 12/14; 86%) of the studies were conducted with breast cancer survivors [30, 31, 33, 34, 60, 63–68, 71].

Six studies (n = 6/14; 43%) examined the association of inflammatory markers (e.g., cytokines, immune cells) and aging-related outcomes [31, 65–68, 71]. In a longitudinal study involving 144 patients with breast cancer aged 50 + , Gilmore et al. reported a correlation between elevated serum levels of interleukin (IL)-6 and soluble tumor necrosis factor-alpha (TNF-alpha) with increased frailty post-chemotherapy [65]. Another study by Gilmore et al. found a positive association between pre-chemotherapy neutrophil to lymphocyte ratio (NLR) and post-chemotherapy frailty in 586 patients with breast cancer [67]. In a longitudinal study with 400 older breast cancer survivors, Carroll et al. found that higher C-reactive protein levels predicted lower self-reported cognition [31]. Belcher et al. found that higher IL-8 levels were associated with worse attention, while higher IL-4 and IL-10 levels were linked to better performance on cognitive measures [66].

Another eight studies (n = 8/14; 57%) examined the association of epigenetic markers, telomere length, and DNA damage with aging-related outcomes [30, 33, 34, 55, 56, 60, 63, 64]. Carroll et al. and Alhareeri et al. found that greater DNA damage and lower telomerase activity were related to worse cognitive function [60, 64]. Yao et al. found an association between epigenetic changes in leukocyte DNA methylome and self-perceived cognitive decline in breast cancer survivors [63]. Gehle et al. and Smitherman et al. found that in 60 childhood cancer survivors, frailty status was associated with a faster pace of epigenetic aging and higher levels of *p16*^*INK4a*^*,* a marker of cellular senescence [55, 56].

## Discussion

This review summarizes the plethora of research published since the 2018 NCI think tank evaluating whether chemotherapy affects cognitive and physical functional outcomes and frailty in cancer survivors [10]. As revealed by this scoping review, a major advance in the field has been the emergence of research findings from several larger longitudinal studies with well-matched comparator groups and relatively high retention rates, allowing for assessing the impact of cancer and chemotherapy over time. As recommended by the think tank, many studies evaluated data from multiple time points along the treatment continuum (i.e., pre-treatment, early treatment phase, shortly after or six months post-chemotherapy to long-term survivorship), allowing for examination of changes over time. Overall, 8/23 (35%), 19/39 (49%), and 5/15 (33%) studies included three or more time points and a comparator group for evaluation of physical function, cognitive function, and frailty changes over time, respectively, providing for assessment of accelerated aging patterns. Cancer survivors with various cancer types were included in the studies identified, although a predominance of studies included breast cancer survivors only. Several studies since the think tank used recommended measures of cognitive function, physical function and frailty, and included recommended usage of objective and self-report measures. Across the studies included in this review, there was consistent evidence of worsening physical function [34, 47, 48, 58, 69, 83], cognitive function [22, 28, 31, 33, 36, 42, 47–49, 59, 66, 68–70, 73, 75, 77, 78, 81], and indicators of frailty [28, 42, 54, 61, 65, 67] in cancer survivors over time after chemotherapy, with greater declines in cancer survivors over time compared to individuals without cancer in 27/32 (84%) of these studies.

Cognitive function was the most commonly examined outcome among the aging-related outcomes chosen for this review. Several studies used International Cancer and Cognition Task Force recommended assessments (Trail-making Test was most widely used), as well as those recommended for inclusion in geriatric assessment for cognitive screening in older adults (MMSE was most commonly used) and neuroscience-based measures [59, 87, 88]. The cognitive domain was most frequently investigated with objective neurocognitive assessment batteries in studies with multiple time points and a comparator group. Longitudinal and cross-sectional studies showed differences in cognitive functional outcomes between cancer survivors and those without cancer, and the highest quality studies included a comparator group of similar age, sex, and educational level. Importantly, cognitive changes were identified in cancer survivors in various age groups addressing recommendations from the think tank to address the changes across the lifespan. Studies demonstrated cognitive functional declines in middle-aged and older adult populations receiving chemotherapy compared to age-matched controls who did not receive chemotherapy, supporting accelerated aging [22, 28, 31, 33, 42, 47, 59, 66, 68–70, 73, 75, 77, 81]. Cross-sectional studies also revealed cognitive functional outcome differences between childhood cancer survivors and age-matched controls [45, 55]. Multiple studies demonstrated that cognitive deficits could persist for even several years after treatment [42, 48, 49, 52]; however, longer-term follow-up data that evaluate survivors 5 to 10 years post-therapy are still needed.

Most studies demonstrated evidence of physical functional declines [19–21, 26, 34, 47, 48, 57, 58, 69, 80, 83]. Physical function was evaluated using patient-reported measures recommended by the 2018 think tank [10], such as Instrumental Activities of Daily Living and self-reported difficulties on physical tasks; the majority of studies utilized validated HRQOL scales (e.g., SF-36) [40, 45, 47–49, 56–58, 79, 83]. Aging-sensitive objective physical performance measures (e.g., Timed Up and Go; gait speed), as recommended by the 2018 think tank, were not frequently utilized to assess physical function. Most longitudinal studies demonstrated physical functional declines, with baseline symptoms, disability, and cognitive function increasing the likelihood of physical functional decline [21, 35]. In studies with several time points that included a comparator group, cancer survivors showed an increased rate of physical functional decline using both patient-reported and objective measures, demonstrating evidence for accelerated aging [34, 47, 48, 58, 69, 83].

The Studies predominantly utilized Fried's frailty criteria and the DAI to measure frailty. Findings consistently pointed to increased frailty among childhood cancer survivors over time and compared to those without cancer, indicating that frailty can be a substantial issue for this population [39, 40, 42, 50, 51, 54–56]. While frailty is usually considered an aging-related condition in older adults [28], recent research suggests frailty characteristics develop following chemotherapy in adults in midlife [40]; confirmatory studies are needed to validate these findings. Similarly, childhood cancer survivors accumulate deficits for years following their cancer diagnosis [54]. Future prospective cohort studies should address frailty trajectories over time in middle-aged adults and pediatric and young adult populations.

Biologic processes linked to functional impairments and decline may be useful biomarkers for understanding how biologic processes change over time with respect to functional decline and for predicting the likelihood of worsening in function. In general, inflammation has been consistently shown to be associated with greater frailty and worse cognitive functional outcomes [31, 65–68, 71] in studies of innate inflammation and specific immune-mediated effector signaling molecules. An avenue of future research in this area is to comprehensively understand how networks of inflammatory processes track over time from pre-treatment, during treatment, and post-treatment in patients with different trajectories of functional decline. Further, it will be important to understand which markers may contrast those with progressive declines with those who improve over time.

Genetics and epigenetics may also help understand the risk of functional decline and accelerated aging. For example, the APOE4 genotype was associated with cognitive decline in patients receiving chemotherapy compared to those receiving hormonal therapy [30]. While these findings need to be validated in larger studies, APOE4, a marker associated with dementia risk, may be a biomarker for cancer-related cognitive decline. Preliminary epigenetic studies have shown that survivors with greater epigenetic aging reported more cognitive impairment than survivors without epigenetic age increases [56, 60, 63, 64]. It will be important to validate these preliminary findings and further understand the functional implications of specific epigenetic signatures closely linked to aging-related phenotypes and accelerated aging in cancer survivors across the lifespan.

### Limitations and gaps

Since our scope was to provide a broad overview of recent research that assessed physical functional and cognitive changes and overall frailty among cancer survivors, it is possible that other studies, especially those with terms not included in our eligibility search criteria, may have been missed. We did not include specific biologic mechanisms as search terms; instead, we reported on biologic mechanisms associated with aging-related outcomes in the manuscripts we identified using the employed search terms. A more comprehensive systematic review of biologic contributors of functional changes across the hallmarks of aging and predictors of worsening function is warranted, as we limited our discussion on biology to studies identified from the search. We did not include “attenuated aging” as a search term because these analyses are usually embedded in studies evaluating “accelerated aging,” which we did include as a search term. Further, we did not search using terms for specific subdomains of cognitive function (e.g., memory), physical function (e.g., balance), and frailty (e.g., fatigue). While our review did reveal findings on functional subdomains identified from the search terms used, since we were not explicit on all subdomains, we likely missed studies that focused on the influence of chemotherapy on specific subdomains.

### Future research directions

To continue to make progress, longitudinal studies that evaluate aging-related outcomes over extended periods from diagnosis to years post-treatment are needed; these studies should integrate multiple time points (e.g., pre-treatment, during treatment, post-treatment, and at several follow-ups) and collect data on differing trajectories of patients over time (e.g., patients who improve, remain stable, decline) to increase understanding of which survivors are resilient or recover over time and which continue to decline. Investigators should consistently report whether the results are statistically significant or clinically meaningful. Research efforts should include diverse cancer types to enhance the generalizability of findings. As the landscape of cancer treatment continues to evolve, there is an urgent need for research examining the impact of new modalities and therapies on accelerated aging in cancer survivors. Future studies should investigate the role of social determinants of health, including socioeconomic status, access to healthcare, and social support, which can provide valuable insights into the broader determinants influencing the aging process in cancer survivors. The effects of specific chemotherapy regimens on aging-related outcomes are still largely unknown; future research should evaluate these effects in prospective studies, and systematic reviews or meta-analyses can be considered when there is a more robust evidence base. Additionally, future studies should delve into the mechanisms associated with accelerated aging. Exploring the molecular and cellular pathways (inflammation, epigenetic changes) related to physical and cognitive functional declines may help identify at-risk patients, monitor their physical and cognitive function over time, and ultimately guide targeted therapeutic strategies to mitigate these aging-related consequences.

### Supplementary Information

Below is the link to the electronic supplementary material.

Supplementary file1 (PDF 373 KB)

## Data Availability

All data generated during this study are included in this published article and its supplementary files.
