# Supplementary material for: A scoping review evaluating physical and cognitive functional outcomes in cancer survivors treated with chemotherapy: charting progress since the 2018 NCI think tank on cancer and aging phenotypes
Source: J Cancer Surviv. 2024 May 14;18(4):1089–130. doi: 10.1007/s11764-024-01589-0 (PMC11324695; doi:10.1007/s11764-024-01589-0)
Supplement: Supplementary file 1 — Supplementary file1 (PDF 373 KB) [file 11764_2024_1589_MOESM1_ESM.pdf]

Preferred Reporting Items for Systematic reviews and Meta-Analyses extension for Scoping Reviews (PRISMA-ScR) Checklist

SECTION	ITEM	PRISMA-ScR CHECKLIST ITEM	REPORTED ON PAGE #
**TITLE**			
Title	1	Identify the report as a scoping review.	1
**ABSTRACT**			
Structured summary	2	Provide a structured summary that includes (as applicable): background, objectives, eligibility criteria, sources of evidence, charting methods, results, and conclusions that relate to the review questions and objectives.	3
**INTRODUCTION**			
Rationale	3	Describe the rationale for the review in the context of what is already known. Explain why the review questions/objectives lend themselves to a scoping review approach.	4
Objectives	4	Provide an explicit statement of the questions and objectives being addressed with reference to their key elements (e.g., population or participants, concepts, and context) or other relevant key elements used to conceptualize the review questions and/or objectives.	5
**METHODS**			
Protocol and registration	5	Indicate whether a review protocol exists; state if and where it can be accessed (e.g., a Web address); and if available, provide registration information, including the registration number.	NA
Eligibility criteria	6	Specify characteristics of the sources of evidence used as eligibility criteria (e.g., years considered, language, and publication status), and provide a rationale.	6&7
Information sources*	7	Describe all information sources in the search (e.g., databases with dates of coverage and contact with authors to identify additional sources), as well as the date the most recent search was executed.	6
Search	8	Present the full electronic search strategy for at least 1 database, including any limits used, such that it could be repeated.	Supplement data
Selection of sources of evidence†	9	State the process for selecting sources of evidence (i.e., screening and eligibility) included in the scoping review.	6
Data charting process‡	10	Describe the methods of charting data from the included sources of evidence (e.g., calibrated forms or forms that have been tested by the team before their use, and whether data charting was done independently or in duplicate) and any processes for obtaining and confirming data from investigators.	7
Data items	11	List and define all variables for which data were sought and any assumptions and simplifications made.	7
Critical appraisal of individual sources of evidence§	12	If done, provide a rationale for conducting a critical appraisal of included sources of evidence; describe the methods used and how this information was used in any data synthesis (if appropriate).	NA
Synthesis of results	13	Describe the methods of handling and summarizing the data that were charted.	7

SECTION	ITEM	PRISMA-ScR CHECKLIST ITEM	REPORTED ON PAGE #
**RESULTS**			
Selection of sources of evidence	14	Give numbers of sources of evidence screened, assessed for eligibility, and included in the review, with reasons for exclusions at each stage, ideally using a flow diagram.	Figure 1
Characteristics of sources of evidence	15	For each source of evidence, present characteristics for which data were charted and provide the citations.	--
Critical appraisal within sources of evidence	16	If done, present data on critical appraisal of included sources of evidence (see item 12).	NA
Results of individual sources of evidence	17	For each included source of evidence, present the relevant data that were charted that relate to the review questions and objectives.	8-11
Synthesis of results	18	Summarize and/or present the charting results as they relate to the review questions and objectives.	9-14
**DISCUSSION**			
Summary of evidence	19	Summarize the main results (including an overview of concepts, themes, and types of evidence available), link to the review questions and objectives, and consider the relevance to key groups.	15-16
Limitations	20	Discuss the limitations of the scoping review process.	18
Conclusions	21	Provide a general interpretation of the results with respect to the review questions and objectives, as well as potential implications and/or next steps.	19
**FUNDING**			
Funding	22	Describe sources of funding for the included sources of evidence, as well as sources of funding for the scoping review. Describe the role of the funders of the scoping review.	1

JBI = Joanna Briggs Institute; PRISMA-ScR = Preferred Reporting Items for Systematic reviews and Meta-Analyses extension for Scoping Reviews.

* Where *sources of evidence* (see second footnote) are compiled from, such as bibliographic databases, social media platforms, and Web sites.

† A more inclusive/heterogeneous term used to account for the different types of evidence or data sources (e.g., quantitative and/or qualitative research, expert opinion, and policy documents) that may be eligible in a scoping review as opposed to only studies. This is not to be confused with *information sources* (see first footnote).

‡ The frameworks by Arksey and O'Malley (6) and Levac and colleagues (7) and the JBI guidance (4, 5) refer to the process of data extraction in a scoping review as data charting.

§ The process of systematically examining research evidence to assess its validity, results, and relevance before using it to inform a decision. This term is used for items 12 and 19 instead of "risk of bias" (which is more applicable to systematic reviews of interventions) to include and acknowledge the various sources of evidence that may be used in a scoping review (e.g., quantitative and/or qualitative research, expert opinion, and policy document).

From: Tricco AC, Lillie E, Zarin W, O'Brien KK, Colquhoun H, Levac D, et al. PRISMA Extension for Scoping Reviews (Prisma's): Checklist and Explanation. Ann Intern Med. 2018;169:467–473. doi: 10.7326/M18-0850.

Web of Science

404 records

Cancer (Title) and Survivor* (Title) and Aging OR Ageing OR Senescence OR Immunosenescence OR Longevit* OR Lifespan* OR Life-span* OR Length-of-Life OR Functional-Declin* OR Frailty OR Frailness OR Debilit* OR Mortalit* OR Fatality-Rate* OR Death-Rate* OR Cause-of-Death OR Causes-of-Death OR Fatal-Outcome* OR Lethal-Outcome* OR Premature-Death* OR Survival-Rate* OR Survival-Curve* OR Survival-Probabilit* OR Survival-Time* OR Survival-Period* OR Geroscien* OR Gero-Scien* OR physical function OR cogni* decline OR cogni* function OR neurocognitive impairment OR cogni* disorders OR cogni* impairment OR cancer-related cognitive impairment (Title) and Article (Document Types)

Embase

354 records

('cancer survivor'/exp OR (Cancer*.ab,ti AND Survivor*.ab,ti) AND ('cognitive aging'/exp OR 'longevity'/exp OR 'national health organization'/exp OR 'frailty'/exp OR 'all cause mortality'/exp OR 'gerontology'/exp OR Aging:ab,ti OR Ageing:ab,ti OR Senescence:ab,ti OR Immunosenescence:ab,ti OR Longevit*:ab,ti OR Lifespan*:ab,ti OR Life-span*:ab,ti OR Length-of-Life:ab,ti OR Functional-Declin*:ab,ti OR Frail*:ab,ti OR Frailness:ab,ti OR Debilit*:ab,ti OR Mortalit*:ab,ti OR Fatality-Rate*:ab,ti OR Death-Rate*:ab,ti OR Cause*-of-Death:ab,ti OR Fatal-Outcome*:ab,ti OR Lethal-Outcome*:ab,ti OR Premature-Death*:ab,ti OR Survival-Rate*:ab,ti OR Survival-Curve*:ab,ti OR Survival-Probabilit*:ab,ti OR Survival-Time*:ab,ti OR Survival-Period*:ab,ti OR Geroscien*:ab,ti OR Gero-Scien*:ab,ti OR 'cognitive defect'/exp OR 'cognition'/exp OR physical-function:ab,ti OR cogni* decline:ab,ti OR cogni*-function:ab,ti OR neurocognitive-impairment:ab,ti OR cogni*-disorders:ab,ti OR cogni*-impairment:ab,ti OR cancer-related-cognitive-impairment:ab,ti)) AND ('article'/it OR 'article in press'/it)

Pubmed

210 records

(Cancer Survivors[Mesh] OR (Cancer*[tiab] AND Survivor*[tiab])) AND ("Aging"[Mesh] OR "Longevity"[Mesh] OR "Aging, Premature"[Mesh] OR "Healthy Aging"[Mesh] OR "National Institute on Aging (U.S.)"[Mesh] OR "Frailty"[Mesh] OR "Mortality"[Mesh] OR "Geroscience"[Mesh] OR Aging[tiab] OR Ageing[tiab] OR Senescence[tiab] OR Immunosenescence[tiab] OR Longevit*[tiab] OR Lifespan*[tiab] OR Life-span*[tiab] OR Length-of-Life[tiab] OR Functional-Decline*[tiab] OR Frailt*[tiab] OR Frailness[tiab] OR Debilit*[tiab] OR Mortalit*[tiab] OR Fatality-Rate*[tiab] OR Death-Rate*[tiab] OR Cause-of-Death[tiab] OR Causes-of-Death[tiab] OR Fatal-Outcome*[tiab] OR Lethal-Outcome*[tiab] OR Premature-Death*[tiab] OR Survival-Rate*[tiab] OR Survival-Curve*[tiab] OR Survival-Probabilit*[tiab] OR Survival-Time*[tiab] OR Survival-Period*[tiab] OR Geroscien*[tiab] OR Gero-Scien*[tiab]) AND ("Cognitive Dysfunction"[Mesh] OR "Cognition"[MeSH] OR physical function[tiab] OR cogni* decline[tiab] OR cogni* function[tiab] OR neurocognitive impairment[tiab] OR cogni* disorders[tiab] OR cogni* impairment[tiab] OR cancer-related cognitive impairment[tiab]))

Limit to English Language

Supplemental Table 1: Cross sectional Studies Examining Physical Function, Cognitive Function, or Frailty Outcomes Among Cancer Survivors (N=16)

Author	Country	Cancer type	Age Group	Sample Size	Assessment Time	Objective	How outcomes were measured	Primary finding
Winters-stone[1] 2019	USA	Breast	60+	N=84	>1-year post chemotherapy	Compare patient-report and objective measures of physical function between older breast cancer survivors and cancer-free controls	Self-report: SF-36 and Late Life Function & Disability Instrument (LLFDI)<br><br>Objective: Short Physical Performance Battery (SPPB)	Patient-reported physical function was significantly lower in breast cancer survivors than controls using SF-36 and the LLFDI.<br><br>Breast cancer survivors had significantly lower SPPB scores compared to controls.
Carroll[2] 2019	USA	Breast	Adults	N=94	3–6 years after chemotherapy	Examine whether indicators of biological aging (Leukocyte DNA damage, PBMC telomerase enzymatic activity) would be related to cognitive function in a cohort of breast cancer survivors	Neuropsychological test battery and self-report	Higher DNA damage and lower telomerase were statistically significantly related to lower executive function scores.<br><br>Lower telomerase activity was associated with worse attention and motor speed.
Henneghan[3] 2020	USA	Breast	Adults<br><br>21 to 65 years	N=65	6 months to 10 years after chemotherapy treatment	Evaluate amyloid beta (Aβ) and tau, biomarkers of neurodegeneration, in relation to cognition in breast cancer survivors	Hopkins Verbal Learning Test<br><br>Trail Making Tests A and B<br><br>Controlled Oral Word Association Test (COWA)	Aβ-42, Aβ-40, tau, and cytokines as features explained significant variance in cognitive function.<br><br>Neurodegenerative biomarkers interact with cytokines to influence

								cognitive functioning in breast cancer survivors following chemotherapy.
Williams[4]<br><br>2020	USA	leukemia	Adults<br><br>21+	N=150	End of chemotherapy course	Examine the effects of chronic lymphocytic leukemia and its treatment on cognitive function	Objective tools:<br><br>NIH Toolbox for the Assessment of Neurological and Behavior Function Cognition (NIH-TB)<br><br>Trail Making Test A/B<br><br>Hopkins Verbal Learning Test<br><br>Controlled Word Association Test	Treated patients reported greater cognitive difficulties than treatment-naive patients but did not perform worse on objective measures.
La Carpia[5]<br><br>2020	Italy	Lymphoma	65+	N=63	Not reported	Compare cognition and function in a group of older long-term survivors from Non-Hodgkins Lymphoma (NHL) and in a corresponding group of non-cancer controls of the same age	Cognition: MMSE<br><br>Rey Auditory Verbal Learning Test,<br><br>Digit span<br><br>Corsi span<br><br>Function: ADL, IADL	MMSE was not significantly different between the groups.<br><br>Lymphoma survivors performed worse than controls in executive functioning.<br><br>A small, statistically significant difference was also observed in verbal memory scores between the two groups.<br><br>NHL survivors performed significantly worse than controls in all functional measures.
Syarif[6]<br><br>2020	Indonesia	Breast	Adults	N=243	Post-chemotherapy	Compare the cognitive function perceived by post-chemotherapy breast cancer survivors,	FACT-Cog	Cognitive function in breast cancer survivors post-chemotherapy was worse compared to survivors who did not

						breast cancer survivors without chemotherapy, and females without cancer		receive chemotherapy and females without cancer.
Smitherman[7]<br>2020	USA	Lymphoma, leukemia, sarcoma, Wilms	Childhood survivors<br>18 to 29 years	N= 60	Median: 5.5 years post chemo	Compare levels of P16 mRNA levels among young adult survivors with frailty	Modified Fried's criteria	Frail survivors, compared with robust survivors, exhibit higher levels of *p16INK4a*.
Delaney[8]<br>2021	USA	Lymphoma, leukemia, sarcoma, CNS	Childhood cancer survivors	N= 1432	≥ 5 years from diagnosis	Examine the incidence of frailty among survivors and risk factors for becoming frail	Fried's frailty criteria	Frailty prevalence increased from 6.2% to 13.6% at 5 years.<br>Risk factors for frailty at follow-up among all survivors included chest radiation, cardiac and neurological conditions, lack of strength training, sedentary lifestyle, and frailty at study entry.
Stelwagen[9]<br>2021	Netherlands	testicular	Adults	N= 66	≥ 20 years post-diagnosis	Assess cognitive impairment in long-term survivors after chemotherapy and compare the results with controls	Cognition: Auditory Verbal Learning Test, Letter Fluency Test, Category Fluency Test, and Trail Making Test	Long-term survivors performed worse on cognitive tests compared to controls.
Williams[10]<br>2022	USA	Hodgkin's Lymphoma	Childhood survivors <21 at diagnosis	N= 2710	>5 years post-diagnosis	Characterize neurocognitive function and social attainment impairments in a cohort of HL survivors relative to their sibling peers	CCSS NCS<br>Function:<br>SF36	Survivors, compared with siblings, exhibited more impairment in neurocognitive function and were more likely to be unemployed and have a personal income of less than $ 20,000.<br>Survivors of HL were at higher risk of impairment in physical function compared with siblings.

Magyari[11]<br>2022	Hungary	HL	Adults	N=118	After chemotherapy	Measure cognitive dysfunction in HL patients and to find potential correlations between patient-related factors, the signs and symptoms of their diseases, or therapeutic factors	CANTAB	52% of all patients showed cognitive impairment.<br>Attention was impaired in 35% of patients, working memory and planning were impaired in 25%, while visual memory was affected in 22%.<br>All three functions showed a significant association with inactive employment status.
Pranikoff[12]<br>2022	USA	Lymphoma, leukemia, sarcoma, CNS	Childhood cancer survivors<br>18–29	N=60	Up to 25 years post diagnosis	Assess frailty in relation to functional and quality-of-life measures	Frailty: Fried frailty phenotype<br>Function: SF-36	Compared to the U.S. population means, survivors reported worse health and functional impairments across SF-36 domains that were more common among survivors with (pre)frailty.<br>Pre-frail survivors (vs non-frail) had lower mean scores for general health, physical function, and overall physical health.
Crouch[13]<br>2022	USA	Breast	60+	N=335	3–8 years post-diagnosis	Examine comorbidities, objective cognitive function, and subjective cognitive function and their relationship with physical functioning	Cognition: self-report: Attentional Function Index<br>Objective: included learning, delayed recall, attention, executive function-working memory, and verbal fluency was assessed using valid and reliable neuropsychological assessments	Comorbidities and worse subjective attention were associated with decreased physical functioning.<br>Objective cognitive function measures were not significantly related to physical functioning.

							Function: Physical Functioning Scale (PF-10; subscale of the SF-36)	
Gehle[14]<br>2023	USA	Multiple	Childhood and young adult cancer survivors	N= 60	At least 3 months post chemotherapy	Determine if epigenetic aging is greater in survivors with physiological evidence of frailt.	Modified Fried's criteria	Compared to non-frail survivors, prefrail and frail survivors had a significantly faster pace of epigenetic aging.
Atteveld[15]<br>2023	Netherlands	Multiple	Childhood survivors<br>18–45 years	N=3996	At least 5 years after cancer diagnosis	Explore risk factors for frailty in a national cohort of Dutch childhood cancer survivors	Modified Fried's criteria	Associated factors with frailty included age at diagnosis between 10–18 years, underweight, cranial irradiation, total body irradiation, cisplatin dose of at least 600 mg/m^2, higher carboplatin doses, hyperthyroidism, bone mineral density, and folic acid deficiency.
Root[16]<br>2023	USA	breast	60+	N= 328	Up to 24 months post-diagnosis	Assess the cognitive performance in survivors and controls utilizing cross-sectional cognitive performance data	Digit Symbol; Trail Making A and B<br>NAB List Learning	Survivors performed lower in learning and memory compared to control.<br>Survivors under 75 years of age, exhibited significantly lower performance in both learning, memory and attention, and processing speed and executive function compared to controls.<br>No differences being observed between older survivors (75+) and controls.

Abbreviations: ADL, activities of daily living; chemo, chemotherapy; CANTAB, Cambridge Neuropsychological Test Automated Battery; CS, cross-sectional; IADL, instrumental activities of daily living; FACT, Functional Assessment of Cancer Therapy; EQ-5D-5L, EuroQol 5 domains; FACT, Functional Assessment of Cancer Therapy; HL, Hodgkin lymphoma; SF-36, Short Form Health Survey; SPPB, Short Physical Performance Battery

Supplemental Table 2: Studies Examining Biologic Measures in Relation to Physical Function, Cognitive Function, or Frailty Outcomes

Author, year, country	Cancer Type and age group	Study Type and Sample Size	Objective	Biologic Measure	Outcome Measurement	Primary Finding
Van der Willik[17] 2018 Netherlands	Breast<br><br>Adults	Cohort N=166	Investigate levels of blood cell–based inflammatory markers in breast cancer survivors on average 20 years after chemotherapy and explored the relation between these markers and global cognitive performance	Systemic inflammation status was assessed by the granulocyte-to-lymphocyte ratio (GLR), platelet-to-lymphocyte ratio (PLR), and systemic immune-inflammation index (SII)	Neuropsychological test battery	Breast cancer survivors had significantly lower general cognitive factor than non-exposed participants from the comparator group.<br><br>The association between higher levels of inflammatory markers and lower general cognitive factor was statistically significant in cancer survivors but not among non-exposed participants.
Carroll[2] 2019 USA	Breast<br><br>Adults	CS N=94	Examine whether indicators of biological aging would be related to cognitive function in a cohort of breast cancer survivors	Leukocyte DNA damage, PBMC telomerase enzymatic activity, PBMC TL, and the inflammatory marker sTNF-RII	Neuropsychological test battery and self-report	Higher DNA damage and lower telomerase were statistically significantly related to lower executive function scores.<br><br>Lower telomerase activity was associated with worse attention and motor speed.
Yao[18] 2019 USA	Breast<br><br>Adults	Cohort N=93	Characterize the changes in leukocyte DNA methylome from pre- to post-chemotherapy<br><br>Examine significant methylation changes with perceived cognitive impairment	Epigenetic changes (CpG, cg16936953)	FACT-Cog	Epigenetic changes (CpG, cg16936953) were significantly associated with cognitive decline in breast cancer patients.
Gilmore[19] 2020 USA	Breast Adult 50+	Cohort N=144	Determine if pre-chemotherapy inflammation is predictive of frailty after chemotherapy	interleukin (IL) 6, and soluble tumor necrosis factor-alpha (TNF)	Frailty assessed using a modified Fried's score	Patients with pre-chemo serum levels of IL-6, sTNFRI, and sTNFRII above the median were significantly frailer after chemotherapy than those with levels below the median.
Alhareeri[20] 2020 USA	Breast<br><br>Adults	Cohort N= 77	Assess the potential relationship between telomere length and development of psychoneurological symptoms	Telomere length	CNS Vital Signs computerized neurocognitive testing system	Chromosomal telomere length was significantly associated with 7 of the 8 cognitive domains evaluated, with the strongest relationship being

						noted for chromosome 17 and the visual memory domain (shorter telomeres; lower scores).
Henneghan[3] 2020 USA	Breast<br><br>Adults	CS N=65	Evaluate amyloid beta (Aβ) and tau, biomarkers of neurodegeneration, in relation to cognition in breast cancer survivors	Amyloid beta (Aβ) and tau, biomarkers of neurodegeneration	Hopkins Verbal Learning Test<br><br>Trail Making Tests A and B<br><br>Controlled Oral Word Association Test (COWA)	Neurodegenerative biomarkers interact with cytokines to influence cognitive functioning in breast cancer survivors following chemotherapy.
Smitherman[7] 2020 USA	Lymphoma, leukemia, sarcoma, Wilms<br><br>Childhood survivors 18 to 29 years	CS<br><br>N= 60	Compare levels of P16 mRNA levels among young adult survivors with frailty, a clinical biomarker of physiologic impairment	P16 mRNA levels	Modified Fried's criteria	Frail survivors, compared with robust survivors, exhibit higher levels of *p16INK4a*.
Van Dyk[21] 2021 USA	Breast<br><br>Older adults 60+	Cohort N=427	Evaluate of the role of *APOE* ε2 in longitudinal cognitive function among older breast cancer survivors and a matched control group	*APOE* ε4 polymorphism	Neuropsychological assessment measured attention, processing speed, executive function, and learning and memory	There was an interaction with genotype for attention, processing speed, and executive functioning domain scores.<br><br>The chemotherapy group with an ε2 allele had higher scores at baseline and maintained higher scores over time compared with those without an ε2 allele, and this protective effect was not seen for other groups.<br><br>There was no effect of ε2 on learning and memory domain score.
Gilmore[22] 2021 USA	Breast Adult 50+	Cohort N=586	Investigate whether pre-chemotherapy levels of cellular markers of inflammation and their change with chemotherapy were associated with post-chemotherapy frailty and frailty that persists up to 6 months after the completion of chemotherapy	Immune cell counts: neutrophil to lymphocyte ratio (NLR)<br><br>lymphocyte to monocyte ratio (LMR)	Frailty assessed using a modified Fried's score	There was a positive association between WBCs and NLR and frailty.<br><br>From pre-chemotherapy to post-chemotherapy, a greater increase in cellular markers of inflammation was significantly

						associated with frailty post-chemotherapy.
Belcher[23]<br>2022<br>USA	Breast<br>50+	Cohort<br>N=519	Evaluate serum cytokine in patients with breast cancer before and after chemo compared with controls<br><br>Assess relationships of cytokine and receptor levels with tests of cognitive function	Serum cytokines (interleukin [IL]–4, 6, 8, 10; tumor necrosis factor [TNF]–α) and soluble receptors [sTNFRI, II])	Attention and processing speed were measured by Rapid Visual Processing (RVP), Backward Counting (BCT), and Trail Making-A (TMT-A) tests	Higher IL-8 significantly associated with worse BCT Higher IL-4 and IL-10 significantly associated with better TMT-A.<br><br>Post chemotherapy, higher IL-8, sTNFRII was significantly associated with worse BCT.
Ahles[24]<br>2022<br>USA	Breast<br>60+	Cohort<br><br>N=328	Determine whether older breast cancer survivors score lower on neuropsychological tests compared to matched non-cancer controls and test the hypotheses that survivors who were APOE ε4 carriers would have the lowest cognitive performance	APOE ε4 carriers	Neuropsychological tests	Breast cancer survivors scored significantly lower on all domains of cognitive function.<br><br>A significant two-way interaction demonstrated that the negative effect of ε4 on cognitive performance was stronger among survivors.
Carroll[25]<br>2023<br>USA	Breast<br>60+	Cohort<br>N=400	Examine longitudinal relationships between levels of C-reactive protein (CRP) and cognition in older breast cancer survivors and noncancer controls	CRP levels	FACT-Cog	Survivors had significantly higher adjusted mean ln-CRP than controls at baseline and 12-, 24-, and 60-month visits.<br><br>Higher adjusted ln-CRP predicted lower participant-reported cognition on subsequent visits among survivors, but not controls.
Gehle[14]<br>2023<br>USA	Multiple<br><br>Childhood survivors<br><br>And AYA	CS<br><br>N= 60	Determine if epigenetic aging is greater in survivors with physiological evidence of frailty	DNA methylation-based epigenetic age	Modified Fried's criteria	Compared to non-frail survivors, prefrail and frail survivors had a significantly faster pace of epigenetic aging.
Rentscher[26]<br>2023<br>USA	Breast<br>60+	Cohort<br>N=89	Examine whether older breast cancer survivors showed greater epigenetic aging than controls and whether epigenetic aging related to functional outcomes	Epigenetic aging using these measures: Horvath, Extrinsic Epigenetic Age, PhenoAge, Grim Age, Dunedin Pace of Aging	Physical function using Medical Outcomes Study Short Form-12	Older breast cancer survivors, particularly those exposed to chemotherapy, showed greater epigenetic aging.<br><br>An older epigenetic age was associated with worse physical function.

AYA, adolescents and young adults; CNS, central nervous system; CS, cross sectional; FACT-Cog, Functional Assessment of Cancer Therapy-Cognitive

1. Winters-Stone KM, Medysky ME, Savin MA. Patient-reported and objectively measured physical function in older breast cancer survivors and cancer-free controls. Journal of Geriatric Oncology 2019;10:311-6.
2. Carroll JE, Van Dyk K, Bower JE, et al. Cognitive performance in survivors of breast cancer and markers of biological aging. Cancer 2019;125:298-306.
3. Henneghan A, Haley AP, Kesler S. Exploring Relationships Among Peripheral Amyloid Beta, Tau, Cytokines, Cognitive Function, and Psychosomatic Symptoms in Breast Cancer Survivors. Biological research for nursing 2020;22:126-38.
4. Williams AM, van Wijngaarden E, Seplaki CL, et al. Cognitive function in patients with chronic lymphocytic leukemia: a cross-sectional study examining effects of disease and treatment. Leuk Lymphoma 2020;61:1627-35.
5. La Carpia D, Liperoti R, Guglielmo M, et al. Cognitive decline in older long-term survivors from Non-Hodgkin Lymphoma: a multicenter cross-sectional study. J Geriatr Oncol 2020;11:790-5.
6. Syarif H, Waluyo A, Afiyanti Y. Cognitive Perception among Post-Chemotherapy, Non-Chemotherapy Breast Cancer Survivors and Non-Cancer. Asian Pacific journal of cancer prevention : APJCP 2021;22:1775-80.
7. Smitherman AB, Wood WA, Mitin N, et al. Accelerated aging among childhood, adolescent, and young adult cancer survivors is evidenced by increased expression of p16(INK4a) and frailty. Cancer 2020;126:4975-83.
8. Delaney A, Howell CR, Krull KR, et al. Progression of Frailty in Survivors of Childhood Cancer: A St. Jude Lifetime Cohort Report. J Natl Cancer Inst 2021;113:1415-21.
9. Stelwagen J, Meuleman AT, Lubberts S, et al. Cognitive impairment in long-term survivors of testicular cancer more than 20 years after treatment. Cancers 2021;13.
10. Williams AM, Mirzaei Salehabadi S, Xing M, et al. Modifiable risk factors for neurocognitive and psychosocial problems after Hodgkin lymphoma. Blood 2022;139:3073-86.
11. Magyari F, Virga I, Simon Z, et al. Assessment of cognitive function in long-term Hodgkin lymphoma survivors, results based on data from a major treatment center in Hungary. Supportive Care in Cancer 2022;30:5249-58.
12. Pranikoff S, Ayer Miller VL, Heiling H, et al. Frail young adult cancer survivors experience poor health-related quality of life. Cancer 2022;128:2375-83.
13. Crouch A, Champion VL, Von Ah D. Comorbidity, cognitive dysfunction, physical functioning, and quality of life in older breast cancer survivors. Supportive Care in Cancer 2022;30:359-66.
14. Gehle SC, Kleissler D, Heiling H, et al. Accelerated epigenetic aging and myopenia in young adult cancer survivors. Cancer Med 2023;12:12149-60.
15. Atteveld JE, de Winter DTC, Pluimakers VG, et al. Frailty and sarcopenia within the earliest national Dutch childhood cancer survivor cohort (DCCSS-LATER): a cross- sectional study. Lancet Healthy Longevity 2023;4:E155-E65.
16. Root JC, Li Y, Schofield E, et al. Cognitive Aging in Older Breast Cancer Survivors. Cancers (Basel) 2023;15.
17. Van Der Willik KD, Koppelmans V, Hauptmann M, Compter A, Ikram MA, Schagen SB. Inflammation markers and cognitive performance in breast cancer survivors 20 years after completion of chemotherapy: A cohort study. Breast Cancer Research 2018;20.
18. Yao S, Hu Q, Kerns S, et al. Impact of chemotherapy for breast cancer on leukocyte DNA methylation landscape and cognitive function: a prospective study. Clinical Epigenetics 2019;11:45.
19. Gilmore N, Kadambi S, Lei L, et al. Associations of inflammation with frailty in patients with breast cancer aged 50 and over receiving chemotherapy. J Geriatr Oncol 2020;11:423-30.
20. Alhareeri AA, Archer KJ, Fu H, et al. Telomere lengths in women treated for breast cancer show associations with chemotherapy, pain symptoms, and cognitive domain measures: a longitudinal study. Breast Cancer Res 2020;22:137.

21. Van Dyk K, Zhou X, Small BJ, et al. Protective Effects of APOE ϵ2 Genotype on Cognition in Older Breast Cancer Survivors: The Thinking and Living with Cancer Study. JNCI Cancer Spectrum 2021;5.
22. Gilmore N, Mohile S, Lei L, et al. The longitudinal relationship between immune cell profiles and frailty in patients with breast cancer receiving chemotherapy. Breast Cancer Research 2021;23:1-11.
23. Belcher EK, Culakova E, Gilmore NJ, et al. Inflammation, Attention, and Processing Speed in Patients With Breast Cancer Before and After Chemotherapy. J Natl Cancer Inst 2022;114:712-21.
24. Ahles TA, Schofield E, Li YL, et al. Relationship between cognitive functioning and frailty in older breast cancer survivors. Journal of Geriatric Oncology 2022;13:27-32.
25. Carroll JE, Nakamura ZM, Small BJ, et al. Elevated C-Reactive Protein and Subsequent Patient-Reported Cognitive Problems in Older Breast Cancer Survivors: The Thinking and Living With Cancer Study. Journal of Clinical Oncology 2023;41:295-306.
26. Rentscher KE, Bethea TN, Zhai WT, et al. Epigenetic aging in older breast cancer survivors and non-cancer controls: preliminary findings from the Thinking and Living with Cancer (TLC) Study. Cancer 2023.
